# Self-encapsulation, or the ‘dripping’ of an elastic rod

**DOI:** 10.1098/rspa.2015.0195

**Published:** 2015-07-08

**Authors:** F. Bosi, D. Misseroni, F. Dal Corso, D. Bigoni

**Affiliations:** DICAM, University of Trento, via Mesiano 77, Trento 38123, Italy

**Keywords:** self-encapsulation, deployable structures, elastica, Eshelby-like force

## Abstract

A rod covering a fixed span is loaded at the middle with a transverse force, such that with increasing load a progressive deflection occurs. After a certain initial deflection, a phenomenon is observed where two points of the rod come in contact with each other. This is defined as the ‘dripping point’ and is when ‘self-encapsulation’ of the elastic rod occurs. Dripping seems at a first glance to be impossible and definitely cannot occur in the presence of ‘ordinary’ constraints (such as simple supports or clamps) at the ends of the span. However, the elastica governs oscillating pendulums, buckling rods and pendant drops, so that a possibility for self-encapsulation might be imagined. This phenomenon is indeed demonstrated (both theoretically and experimentally) to occur when at least one of the constraints at the ends of the rod is a sliding sleeve. This mechanical device generates a configurational force, causing the dripping of the rod, in a fully elastic set-up.

## 1. Introduction

**Figure 1.**
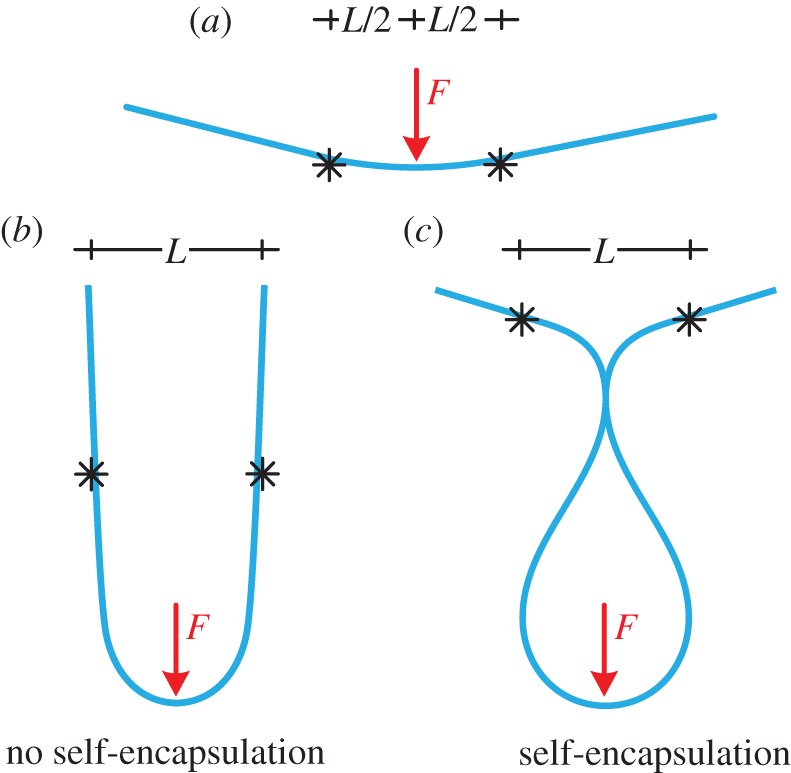
Schematics of the self-encapsulation, or the dripping, of an elastic rod. (*a*) An elastic rod is loaded at midspan between two constraints at fixed distance *L*. (*b*) Self-encapsulation does not occur (as in the case when the two constraints are simple supports). (*c*) Self-encapsulation, which may be re-phrased as the ‘dripping of an elastic rod’, occurs. This requires the use of a ‘non-standard’ constraint, such as a sliding sleeve.

Is it possible to load an elastic rod with a transverse force at midspan between two constraints at fixed distance *L* in order to reach a closed deformation loop (figure 1)? In other words, is self-encapsulation or ‘dripping’ of an elastic rod possible?^1^ Although this problem may seem of academic interest only, it has connections to micro- or nano-fabrication technologies for deployable structures used for instance in sensor technology. In this field of application, self-assembly can be achieved through magnetic forces [2], while a self-folding spherical structure—the so-called ‘buckliball’—has been invented [3] and a dynamic self-encapsulation technique for a thin plate and a rod has already been pointed out [4,5]. In the former case only a reduction in the volume of a sphere is achieved, while in the latter self-encapsulation is obtained, but as a result of both dynamic effects and capillary forces, which are related to the presence of a liquid droplet attached to the rod. Therefore, the self-encapsulation problem as addressed in this article has never been challenged before and indeed may seem impossible at a first glance. However, the fact that the differential equation of the elastica not only governs the oscillation of a simple pendulum and the deflection of an elastic rod, but also the shape of a droplet [6] should stimulate the belief that dripping of an elastic rod could be possible.

**Figure 2.**
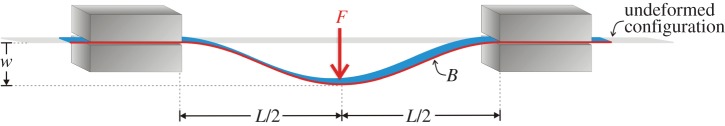
Sketch of the structure showing dripping of an elastic rod. An elastic planar rod, of bending stiffness *B*, is constrained with a frictionless sliding sleeve at both ends. The distance between the two constraints, *L* is fixed, but the rod between the two constraints has a variable length, function of the transverse load *F* applied at midspan.

The key point in solving the above-formulated self-encapsulation problem lies in the choice of the constraints at the ends of the span, namely, a couple of (perfectly smooth) sliding sleeves (figure 2). In the presence of a bending moment, the sliding sleeve has been shown to generate an ‘Eshelby-like’ or ‘configurational’ force [7–10], which provides the longitudinal compression needed to produce dripping. A similar mechanical system has already been studied, but without considering the configurational force [11,12], and thus dripping has remained undiscovered.

Self-encapsulation is demonstrated (theoretically with a fully nonlinear solution of the elastica [13,14] and experimentally^2^ in a qualitative and quantitative way) to occur in the mechanical system shown in figure 2. This structure exhibits a load reversal in the load/deflection diagram, so that three kinds of experiments were performed to induce the dripping of the rod.

**Figure 3.**
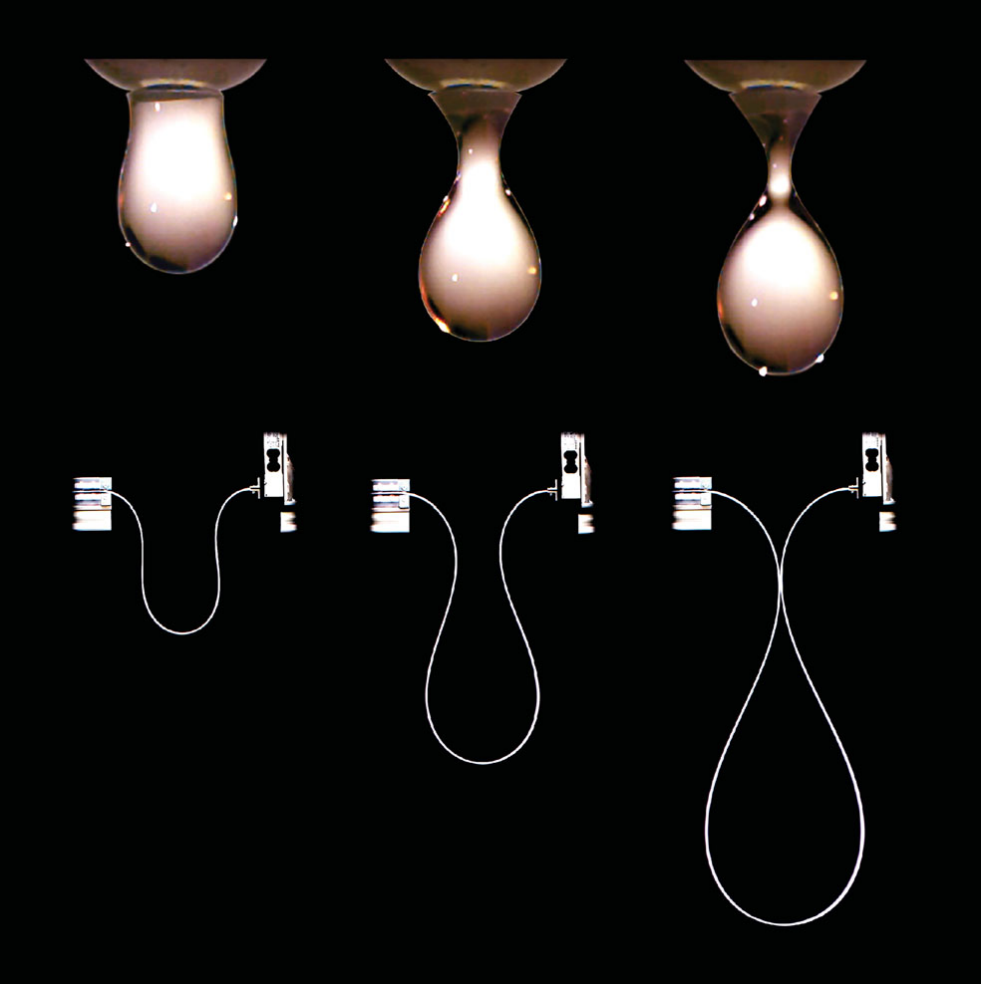
The dripping of an elastic rod. Upper part: the progressive formation of a drop (photos taken with a Photron Fastcam SA5 model 775K-C2 at 10 000 fps). Lower part: the self-encapsulating rod under quasi-static load (photos taken with a Nikon D200 with a AF Nikkor 18–35 mm). Note the analogy in the shapes of the drop and of the rod.

In particular, the experimental verification of the symmetric solution obtained in §2 requires imposition of the full displacement of the midspan (so that the configurations assumed by the system are stable and dripping can be obtained without departures from symmetry). This experiment was performed only in a qualitative way (to produce the photos shown in figure 3, lower part, where a series of quasi-statically deformed shapes of the rod showing self-encapsulation is reported and compared with the shapes of a forming drop, upper part), because it was considered trivial, while experiments were designed not only to confirm the analytical solution, but also to demonstrate that self-encapsulation is a robust phenomenon, occurring even when the symmetry conditions on which the solution is based are perturbed. Therefore, other qualitative experiments were performed taking advantage of the fact that the structure shows a load reversal, which allows the rod to rest in a certain deformed (unstable) configuration without any applied external load. In these experiments, this configuration was induced by imposing a displacement at midspan of the rod and then perturbing it to trigger a spontaneous dynamics that causes the rod to take the shape of a progressively forming drop, culminating at the dripping point (self-encapsulation), and continuing with the enlargement of the drop and a break of symmetry, as illustrated in the video available in the electronic supplementary material (and also at http://ssmg.unitn.it). Finally, quantitative (quasi-static) experiments were performed in which only the vertical displacement at the midspan was imposed with a testing machine (and the corresponding load measured). In this way, the analytical solution is rigorously followed only until the force reversal, when the symmetry is broken and an additional force is generated at the device imposing the displacement, see §3a(i). Even in this case dripping is obtained and the perturbation induced by the new generated force is shown not to significantly affect the load/displacement diagram predicted by the symmetric solution. It can be therefore concluded from the experiments that the analytical solution is fully confirmed and that dripping is a robust phenomenon that occurs even when the ideal conditions assumed to obtain the analytical solution are perturbed.

It is important to remark that the self-encapsulation (and also the dripping) occurs in an elastic and frictionless system, so that all presented structural transformations are fully repeatable and without hysteresis, opening a new perspective in reversible and tuneable encapsulation.

## 2. The equilibrium configurations and the self-encapsulation point

**Figure 4.**
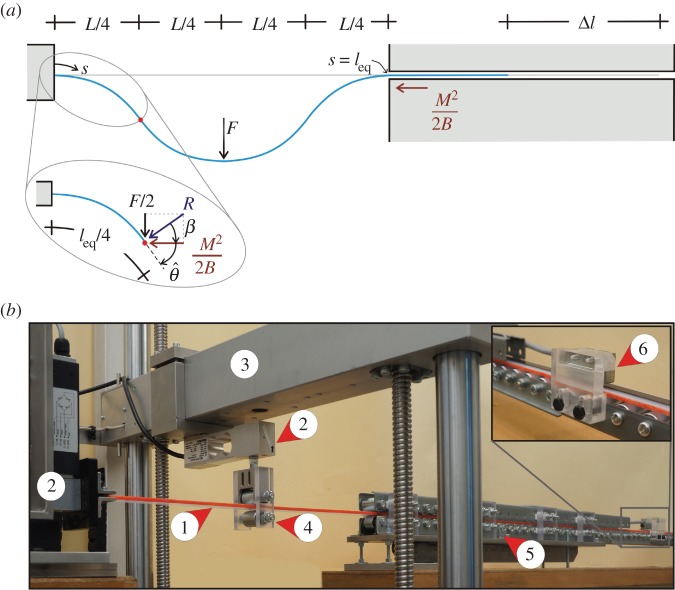
(a) Scheme used to investigate the symmetric solution of the elastic system sketched in figure 2 and loaded at the midspan with a concentrated transverse force *F*. Loading the structure generates the compressive configurational force *M*^2^/2*B*, acting at the sliding sleeve in the axial direction. Exploiting symmetry, the structure can be divided into four rods of equal length *l*_eq_/4 subject to the transverse load *F*/2 and to the axial configurational force *M*^2^/2*B*. (*b*) The experimental set-up for quasi-static experiments comprising elastic rod (1), load cells (2), movable crosshead (3), bilateral roller (4), sliding sleeve (5) and displacement transducer (6).

Considering symmetric equilibrium configurations, the planar rod constrained by a couple of sliding sleeves at both ends, figure 2, is here analysed by replacing the left sliding sleeve with a clamp, figure 4*a*. The presence of a clamp on the left end is also representative of the quantitative experimental test described in §3a and performed to measure the Eshelby-like force through a load cell.

With reference to an inextensible Euler–Bernoulli model for the planar rod, the rotation field of the rod's axis *θ*(*s*) represents the relevant kinematic field, function of the curvilinear coordinate $s\in [0,\bar{l}]$, where $\bar{l}$ is the total length of the rod of bending stiffness *B*, with $\bar{l}\geq L$, where *L* is the given distance between the clamp and the sliding sleeve. When a transverse load *F* is applied at the rod midspan, the total potential energy W of the elastic system can be written as

2.1 $$W(\theta (s),l_{\mathrm{out}})=\int_{0}^{l_{\mathrm{out}}}B\frac{[\theta^{\prime }(s){]}^{2}}{2}\;ds-F\int_{0}^{l_{\mathrm{out}}/2}\sin\theta (s)\;ds+V\int_{0}^{l_{\mathrm{out}}}\sin\theta (s)\;ds,$$

where a prime denotes the spatial derivative, and $l_{\mathrm{out}}\in [L,\bar{l})$ is the length of the deformed elastic planar rod between the two constraints (the clamp and the sliding sleeve). Note that the rotation is null within the sliding sleeve, *θ*(*s*)=0 with $s\in [l_{\mathrm{out}},\bar{l})$. The last term in the total potential energy W represents the work done by the vertical upward reaction *V* at the sliding sleeve, which has to be null for unmovable constraint so that

2.2 $$\int_{0}^{l_{\mathrm{out}}}\sin\theta (s)\;ds=0,$$

an equation revealing that *V* acts as a Lagrange multiplier.

The length *l*_out_ and the rotation field *θ*(*s*) satisfy the following geometrical condition expressing *L*, the distance between the two constraints,

2.3 $$L=\int_{0}^{l_{\mathrm{out}}}\cos\theta (s)\;ds.$$

For a given load *F*, the equations governing the equilibrium configuration can be obtained by introducing a small parameter *ϵ* to describe the variations *θ*_var_(*s*) and *l*_var_ of the equilibrium configuration in the rotation field *θ*_eq_(*s*) and the rod's length between the two constraints *l*_eq_ as

2.4 $$\theta (s,\epsilon )=\theta_{\mathrm{eq}}(s)+\epsilon \theta_{\mathrm{var}}(s)\;\mathrm{and}\;l_{\mathrm{out}}(\epsilon )=l_{\mathrm{eq}}+\epsilon l_{\mathrm{var}}.$$

A first-order Taylor series expansion of *θ*(*l*_out_) in *ϵ* and of the geometrical condition (2.3), together with the boundary conditions at the sliding sleeve, namely *θ*_eq_(*l*_eq_)=0 and *θ*(*l*_out_)=0, leads to the following compatibility equations:

2.5 $$\theta_{\mathrm{var}}(l_{\mathrm{eq}})=-\theta_{\mathrm{eq}}^{\prime }(l_{\mathrm{eq}})l_{\mathrm{var}},\;l_{\mathrm{var}}=\int_{0}^{l_{\mathrm{eq}}}\sin\theta_{\mathrm{eq}}(s)\theta_{\mathrm{var}}(s)\;ds.$$

Restricting now attention to symmetric equilibrium configurations (figure 4*a*; effects of lack of symmetry are considered in §3a), it follows that *V* =*F*/2 and the rotation field has the following symmetry property

2.6 $$\theta_{\mathrm{eq}}(s)=-\theta_{\mathrm{eq}}(l_{\mathrm{eq}}-s),\;\text{for }s\in \left[0,\frac{l_{\mathrm{eq}}}{2}\right],$$

so that, an account of the boundary condition at the clamp *θ*_eq_(0)=0 and of the compatibility condition (2.5)_1_, yields the first variation of the functional W as

2.7 $$\begin{array}{rl} \delta_{\epsilon }W & =-\int_{0}^{l_{\mathrm{eq}}/2}\left[B\theta_{\mathrm{eq}}^{\prime \prime }+\frac{F}{2}\cos\theta_{\mathrm{eq}}(s)\right]\theta_{\mathrm{var}}(s)\;ds\\ & \;-\int_{l_{\mathrm{eq}}/2}^{l_{\mathrm{eq}}}\left[B\theta_{\mathrm{eq}}^{\prime \prime }-\frac{F}{2}\cos\theta_{\mathrm{eq}}(s)\right]\theta_{\mathrm{var}}(s)\;ds-\frac{B}{2}\theta_{\mathrm{eq}}^{\prime }(0{)}^{2}l_{\mathrm{var}}. \end{array}$$

Imposing the vanishing of the first variation of the total potential energy $\delta_{\epsilon }W$ for every admissible *θ*_var_(*s*) provides the governing equation of the elastica for the deflected rod

2.8 $$\begin{array}{rl} & B\theta_{\mathrm{eq}}^{\prime \prime }(s)+\frac{F}{2}\cos\theta_{\mathrm{eq}}(s)+\frac{B\theta_{\mathrm{eq}}^{\prime }(0{)}^{2}}{2}\sin\theta_{\mathrm{eq}}(s)=0,\;s\in \left(0,\frac{l_{\mathrm{eq}}}{2}\right)\\ \;\mathrm{and}\;\; & B\theta_{\mathrm{eq}}^{\prime \prime }(s)-\frac{F}{2}\cos\theta_{\mathrm{eq}}(s)+\frac{B\theta_{\mathrm{eq}}^{\prime }(0{)}^{2}}{2}\sin\theta_{\mathrm{eq}}(s)=0,\;s\in \left(\frac{l_{\mathrm{eq}}}{2},l_{\mathrm{eq}}\right), \end{array}\}$$

subject to the boundary conditions *θ*_eq_(0)=*θ*_eq_(*l*_eq_/2)=*θ*_eq_(*l*_eq_)=0. Assuming the bending moment *M* to be proportional to the curvature (*M*(*s*)=*Bθ*′_eq_(*s*)), the equilibrium equations (2.8) include the configurational or Eshelby-like force [7]

2.9 $$\frac{M^{2}}{2B}=\frac{B\theta_{\mathrm{eq}}^{\prime }(0{)}^{2}}{2},$$

which is crucial for achieving self-encapsulation. Of course, the differential problem (2.8) governs the equilibrium configuration only up to the dripping point, or the self-encapsulation.

Let us restrict the attention to the first half of the deflected rod. By introducing the load parameter

2.10 $$\gamma^{2}=\frac{R}{B},\;\text{with}\;R=\sqrt{{\left(\frac{F}{2}\right)}^{2}+{\left({\frac{M}{2B}}^{2}\right)}^{2}},$$

and the auxiliary angle *ψ*(*s*)=*θ*_eq_(*s*)+*β*, where *β* is the inclination of the resultant *R* with respect to the straight undeformed rod's axis, the differential problem (2.8)_1_ can be rewritten as

2.11 $$\begin{array}{rl} & \psi^{\prime \prime }(s)+\gamma^{2}\sin\psi (s)=0,\;s\in \left[0,\frac{l_{\mathrm{eq}}}{2}\right]\\ \;\mathrm{and}\;\; & \psi (0)=\psi \left(\frac{l_{\mathrm{eq}}}{2}\right)=\beta . \end{array}\}$$

Owing to the symmetry of the equilibrium configuration, the inflection point is located at the quarter point of the deflected part of the rod (*s*=*l*_eq_/4),

2.12 $$\theta_{\mathrm{eq}}^{\prime }\left(\frac{l_{\mathrm{eq}}}{4}\right)=0\;\mathrm{and}\;\psi^{\prime }\left(\frac{l_{\mathrm{eq}}}{4}\right)=0$$

and, defining the rotation in such a point as $\hat{\theta }=\theta (l_{\mathrm{eq}}/4)$, it follows that $\psi (l_{\mathrm{eq}}/4)=\hat{\psi }=\hat{\theta }+\beta$, so that integration of equation (2.11)_1_ yields

2.13 $$\psi^{\prime }(s)=\pm \gamma \sqrt{2(\cos\psi (s)-\cos\hat{\psi })},$$

where the ‘+’ (or ‘−’) sign holds for *s*∈[0,*l*_eq_/4] (or for *s*∈[*l*_eq_/4,*l*_eq_/2]). Introducing the following change of variables:

2.14 $$\eta =\sin\frac{\hat{\psi }}{2}\;\mathrm{and}\;\eta \sin\omega (s)=\sin\frac{\psi (s)}{2},$$

differential equation (2.13) can be integrated to obtain the relationship between the load parameter *γ* and the angles $\hat{\theta }$ and *β* as

2.15 $$\gamma l_{\mathrm{eq}}=4[K(\eta )-K(\omega_{\beta },\eta )],$$

where $\omega_{\beta }=\arcsin((1/\eta )\sin(\beta /2))$ and $K(\omega_{\beta },\eta )$ is the incomplete elliptic integral of the first kind. Equation (2.15) contains as unknowns the configurational force *M*^2^/2*B* (present in the parameter *γ*) and the length of the rod in its reference configuration *l*_eq_. Inverting equation (2.10), the former can be expressed as

2.16 $$\frac{M^{2}}{2B}=2B\gamma^{2}\left(\eta^{2}-\sin^{2}\frac{\beta }{2}\right),$$

and the applied load *F* can be expressed as

2.17 $$F=\mathrm{sgn}\left[\frac{\pi }{2}-\hat{\theta }\right]2B\gamma^{2}\sqrt{1-4{\left(\eta^{2}-\sin^{2}\frac{\beta }{2}\right)}^{2}},$$

where the function sgn (defined as sgn[*x*]=|*x*|/*x* ∀ *x*∈Re\0 and sign[0]=0) has been introduced.

Expression (2.17) for the load *F* makes evident that *F*=0 at $\hat{\theta }=\pi /2$, which defines the load reversal. Equation (2.17) becomes explicit once the equations describing the shape of the elastica are obtained. In particular, from equation (2.14)_2_ the rotational field for *s*∈[0,*l*_eq_/2] can be obtained as

2.18 $$\theta_{\mathrm{eq}}(s)=2\arcsin[\eta \;\mathrm{sn}(\gamma s+K(\omega_{\beta },\eta ),\eta )]-\beta ,$$

while the axial and transverse positions for *s*∈[0,*l*_eq_/2] can be calculated from an integration of the kinematic fields

2.19 $$x_{1}(s)=\int_{0}^{s}\cos\theta_{\mathrm{eq}}(\tau )\;d\tau \;\mathrm{and}\;x_{2}(s)=\int_{0}^{s}\sin\theta_{\mathrm{eq}}(\tau )\;d\tau ,$$

in the form

2.20 $$\begin{array}{rl} x_{1}(s) & =\sin\beta \left[-\frac{2\eta }{\gamma }\;\mathrm{cn}(\gamma s+K(\omega_{\beta },\eta ),\eta )+\frac{2\eta }{\gamma }\;\mathrm{cn}(K(\omega_{\beta },\eta ),\eta )\right]\\ & \;+\cos\beta \left\{-s+\frac{2}{\gamma }[E[\mathrm{am}(\gamma s+K(\omega_{\beta },\eta ),\eta ),\eta ]-E[\mathrm{am}(K(\omega_{\beta },\eta ),\eta ),\eta ]]\right\}\\ \;\mathrm{and}\;\;x_{2}(s) & =\cos\beta \left[-\frac{2\eta }{\gamma }\;\mathrm{cn}(\gamma s+K(\omega_{\beta },\eta ),\eta )+\frac{2\eta }{\gamma }\;\mathrm{cn}(K(\omega_{\beta },\eta ),\eta )\right]\\ & \;-\sin\beta \left\{-s+\frac{2}{\gamma }[E[\mathrm{am}(\gamma s+K(\omega_{\beta },\eta ),\eta ),\eta ]-E[\mathrm{am}(K(\omega_{\beta },\eta ),\eta ),\eta ]]\right\}. \end{array}\}$$

Employing symmetry (figure 4), the relation *x*_1_(*l*_eq_/4)=*L*/4 can be written using equation (2.15) as

2.21 $$\begin{array}{rl} \gamma & =\frac{4}{L}\{\cos\beta [K(\omega_{\beta },\eta )-K(\eta )+2[E[\mathrm{am}(K(\eta ),\eta ),\eta ]-E[\mathrm{am}(K(\omega_{\beta },\eta ),\eta ),\eta ]]]\\ & \;-2\eta \sin\beta [\mathrm{cn}(K(\eta ),\eta )-\mathrm{cn}(K(\omega_{\beta },\eta ),\eta )]\}, \end{array}$$

so that the relationship between the dimensionless applied transverse force *FL*^2^/*B* and the parameters *η* (function of $\hat{\theta }$) and *β* is finally obtained from equation (2.17) as

2.22 $$\begin{array}{rl} \frac{FL^{2}}{B} & =32\;\mathrm{sgn}[\pi /2-\hat{\theta }]\sqrt{1-4{\left(\eta^{2}-\sin^{2}\frac{\beta }{2}\right)}^{2}}\{\cos\beta [K(\omega_{\beta },\eta )-K(\eta )+2[E[\mathrm{am}(K(\eta ),\eta ),\eta ]\\ & \;{-E[\mathrm{am}(K(\omega_{\beta },\eta ),\eta ),\eta ]]]-2\eta \sin\beta [\mathrm{cn}(K(\eta ),\eta )-\mathrm{cn}(K(\omega_{\beta },\eta ),\eta )]\}}^{2}, \end{array}$$

while the dimensionless configurational force, accounting for expression (2.16), becomes

2.23 $$\begin{array}{rl} \frac{M^{2}L^{2}}{2B^{2}} & =32\left(\eta^{2}-\sin^{2}\frac{\beta }{2}\right)\{\cos\beta [K(\omega_{\beta },\eta )-K(\eta )+2[E[\mathrm{am}(K(\eta ),\eta ),\eta ]\\ & \;-E[\mathrm{am}(K(\omega_{\beta },\eta ),\eta ),\eta ]]]-2\eta \sin\beta [\mathrm{cn}(K(\eta ),\eta )-\mathrm{cn}(K(\omega_{\beta },\eta ),\eta )]{\}}^{2}. \end{array}$$

Furthermore, the dimensionless length Δ*l*/*L*=*l*_eq_/*L*−1, measuring the amount of elastic rod slipping into the sliding sleeve, can be calculated, according to equations (2.15) and (2.21), to be

2.24 $$\frac{\Delta l}{L}=\frac{K(\eta )-K(\omega_{\beta },\eta )}{\gamma L}-1.$$

Finally, the midspan deflection of the structure, *w*=*x*_2_(*l*_eq_/2), can be written as

2.25 $$w=\frac{1}{\gamma }\{2\eta \cos\beta \cos(\omega_{\beta })+\sin\beta [2E(\omega_{\beta },\eta )-2E(\eta )+K(\eta )-K(\omega_{\beta },\eta )]\},$$

where *γ* is defined by equation (2.21). Equations (2.22), (2.23), (2.24) and (2.25) are all functions of the two parameters *η* and *β*, that can be solved from either relations $\sin\beta =F/(2B\gamma^{2})$ or $\cos\beta =M^{2}/(2B^{2}\gamma^{2})$. Substituting the former expression into the definition of *F*, equation (2.17), yields

2.26 $$\sin\beta =\sqrt{1-4{\left(\eta^{2}-\sin^{2}\frac{\beta }{2}\right)}^{2}}$$

and therefore, using the double-angle formulae and the change of variable (2.14)_1_, the two relations between $\hat{\theta }$ and *β* follow

2.27 $$\sin^{2}\left(\frac{\hat{\theta }+\beta }{2}\right)=\frac{1}{2}\;\mathrm{and}\;\sin^{2}\left(\frac{\hat{\theta }+\beta }{2}\right)=2\sin^{2}\left(\frac{\beta }{2}\right)-\frac{1}{2},$$

where the latter has no physical meaning as it requires $\hat{\theta }<0$, whereas the former is simplified in

2.28 $$\hat{\theta }+\beta =\frac{\pi }{2},$$

showing that for every load *F*, the resultant *R* is always perpendicular to the deformed rod's axis at *s*=*l*_eq_/4.

**Figure 5.**
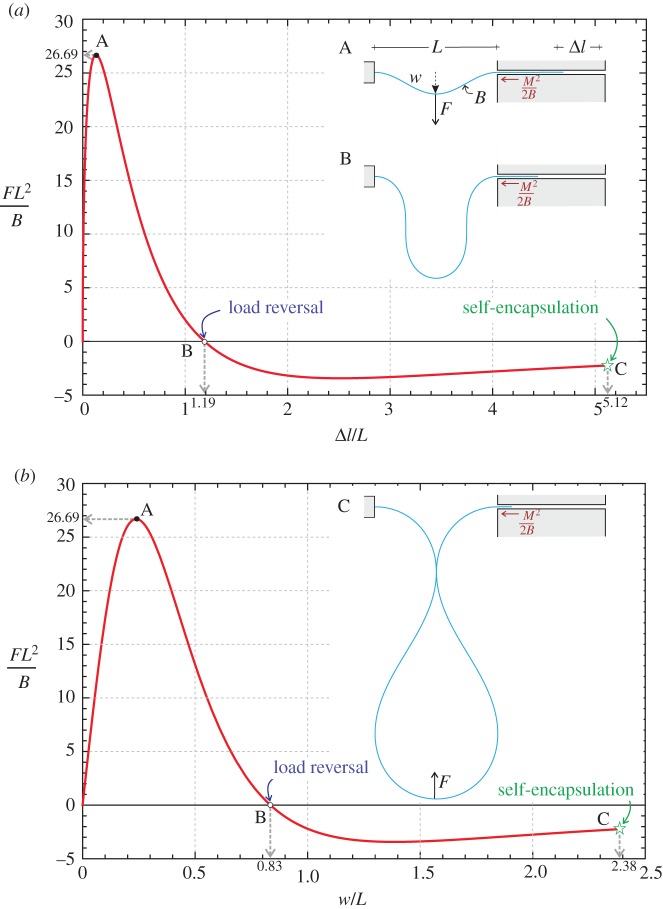
Equilibrium path of the structure sketched in the inset subjected to a concentrated transverse load *F* and deformed symmetrically. The dimensionless length measuring the amount of elastic rod slipping into the sliding sleeve, Δ*l*/*L*, (*a*) and the dimensionless midspan deflection *w*/*l* (*b*) are reported versus the dimensionless applied load *FL*^2^/*B*. The characteristic points of maximum load, load reversal and self-encapsulation are marked on the curves with the letters A, B and C, respectively. Deformed shapes of the elastica are reported in the insets.

The loading path of the elastic rod (deformed symmetrically) is reported in figure 5 in terms of dimensionless applied transverse force *FL*^2^/*B* as a function of the dimensionless length Δ*l*/*L*, which is the length of rod sliding out of the sleeve, and as a function of the midspan dimensionless deflection *w*/*L*.

From figure 5, it can be noted that:

— the maximum load *F*^max^ is
  2.29 $$F^{\max}\approx 26.69\frac{B}{L^{2}},$$
  while the incorrect result calculated in [11], without considering the configurational force, is 64*B*/*L*^2^ giving a overestimation of the load-carrying capacity of 2.4 times;
— the structural system displays a softening behaviour, unstable for imposed dead load, so that a *force reversal* occurs for Δ*l*≈1.19*L* or, equivalently, when *w*≈0.83*L* and $\hat{\theta }=90^{\circ }$; and
— self-encapsulation or the dripping point is reached when $\hat{\theta }\approx 121.24^{\circ }$, Δ*l*≈5.12*L*, *w*≈2.38*L*, *FL*^2^/*B*≈−2.24 and *M*^2^*L*^2^/2*B*^2^≈1.85.

## 3. The experimental proof of self-encapsulation and dripping

The solution derived in §2 for dripping displays softening and a force reversal, so that it is unstable for applied dead load at midspan and, to check its validity, vertical displacement and null horizontal displacement and rotation have to be imposed at midspan. Qualitative tests of this type were performed at the Instabilities Laboratory of the University of Trento (http://ssmg.unitn.it/), showing self-encapsulation with shapes of the elastica in close agreement with the theoretical prediction (figure 3, lower part). However, rather than continuing with this approach, experiments on proof-of-concept structures were designed (according to the scheme of figure 4*a*, in which it is also possible to measure the horizontal force at the clamp) and used to perform the two types of experiment presented below, both aimed at proving that dripping is a robust phenomenon, occurring even when the symmetric solution derived in §2 is not fully applicable. The performed tests were quasi-static experiments, in which the vertical displacement has been imposed at midspan with a testing machine, and dynamic experiments, in which the elastic rod is brought at the inversion load point and left free of dripping in a dynamical motion where the mass of the rod plays a role.

### (a) Quasi-static experiments

In the quasi-static experiments, only the vertical component of the displacement at midspan of the system shown in figure 4*b* is prescribed (horizontal displacement and rotation have left free) and the vertical load is measured (together with the horizontal force at the clamp). Under this condition, the symmetric solution is stable only until the force reversal point, at which a symmetry breaking is expected to occur.

#### (i) Equilibrium equations under non-symmetric conditions

For non-symmetric equilibrium configurations, the vertical reaction *V* is unknown and, in addition to the variations in the rotation field and in the rod's length comprised between the two constraints, equation (2.4), the variation l*_var_ in the length of the rod l*_eq_ between the clamp and the midspan at equilibrium is introduced,

3.1 $$l_{\mathrm{out}}^{*}=l_{\mathrm{eq}}^{*}+\epsilon l_{\mathrm{var}}^{*}.$$

From the geometrical constraint

3.2 $$\frac{L}{2}=\int_{0}^{l_{\mathrm{out}}^{*}}\cos\theta (s)\;ds,$$

the variation l*_var_ can be obtained as

3.3 $$l_{\mathrm{var}}^{*}=\frac{1}{\cos\theta_{\mathrm{eq}}(l_{\mathrm{eq}}^{*})}\int_{0}^{l_{\mathrm{eq}}^{*}}\sin\theta_{\mathrm{eq}}(s)\theta_{\mathrm{var}}(s)\;ds,$$

so that, from the vanishing of the first variation of the total potential energy, the following equilibrium equations are obtained:

3.4 $$\begin{array}{rl} & B\theta_{\mathrm{eq}}^{\prime \prime }(s)+(F-V)\cos\theta_{\mathrm{eq}}(s)+\left[\frac{B\theta_{\mathrm{eq}}^{\prime }(l_{\mathrm{eq}}{)}^{2}}{2}+F\tan\theta_{\mathrm{eq}}(l_{\mathrm{eq}}^{*})\right]\sin\theta_{\mathrm{eq}}(s)=0,\;s\in (0,l_{\mathrm{eq}}^{*})\\ \;\mathrm{and}\;\; & B\theta_{\mathrm{eq}}^{\prime \prime }(s)-V\cos\theta_{\mathrm{eq}}(s)+\frac{B\theta_{\mathrm{eq}}^{\prime }(l_{\mathrm{eq}}{)}^{2}}{2}\sin\theta_{\mathrm{eq}}(s)=0,\;s\in (l_{\mathrm{eq}}^{*},l_{\mathrm{eq}}), \end{array}\}$$

subject to the boundary conditions *θ*_eq_(0)=*θ*_eq_(*l*_eq_)=0.

Note that $F\tan\theta_{\mathrm{eq}}(l_{\mathrm{eq}}^{*})$ is a new horizontal force generated at midspan and related to the fact that the constraint leaves the possibility of horizontal displacement, so that this force shares similarities with the configurational force provided by the sliding sleeve, which is also related to the possibility of horizontal free sliding.

When symmetry applies

3.5 $$l_{\mathrm{eq}}^{*}=\frac{l_{\mathrm{eq}}}{2},\;V=\frac{F}{2}\;\mathrm{and}\;\theta (l_{\mathrm{eq}}^{*})=0,$$

the equilibrium equations (3.4) reduce to equations (2.8) and the ‘extra’ horizontal force vanishes.

After the force reversal in the load/displacement diagram (figure 5), the symmetric configuration assumed for the solution in §2 is certainly unstable, as an upward force is applied to the bottom of a rod shaped as a forming drop. When symmetry is broken the equilibrium equations (3.4) apply, but finding an analytical solution to this problem falls beyond the scopes of this article. Experiments show that the symmetry breaking occurs in reality without precluding dripping.

#### (ii) The design of the structure

**Figure 6.**
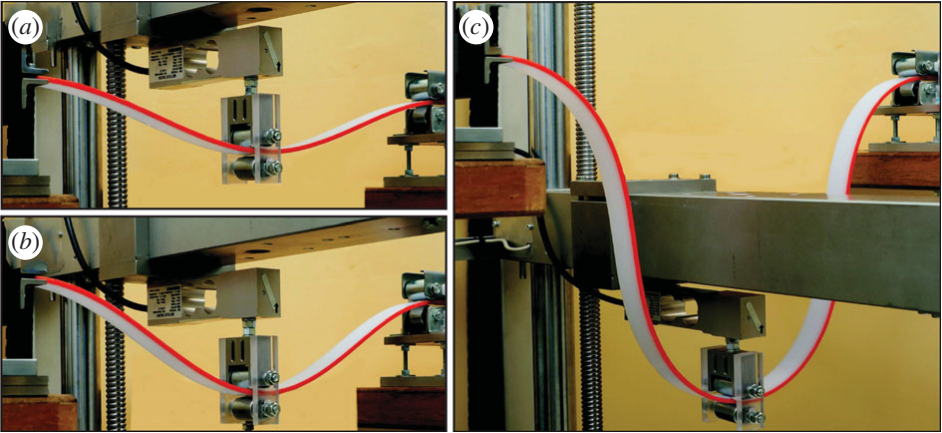
A sequence of photos during an experiment performed on the structure loaded at midspan by imposing displacement with a testing machine. In the three pictures (*a*–*c*) the imposed vertical displacement at the midspan is *w*= {0.16;0.26;0.79}*L*, corresponding to the following measured values of load *F*= {21.54;25.55;0.79}*B*/*L*^2^ and lengths Δ*l*={0.06;0.15;1.15}*L*.

The model structure used for the experiments was loaded by imposing with a bilateral roller (realized with two roller bearings from Misumi Europe, Press-Fit Straight Type, 20 mm in diameter and 25 mm in length) a prescribed vertical displacement at the midspan (through an MIDI 10 load frame from Messphysik), while the vertical reaction force *F* on the roller was measured with a MT1041-R.C. 500 N load cell (from Mettler; figures 4*b* and 6).

Tests were performed using three rods of different thickness of the cross section (*h*={1.9;2.85;3.85} mm), but having the same length (1600 mm) and width (*b*=24.9 mm), and all made in solid polycarbonate (white 2099 Makrolon UV from Bayer, elastic modulus 2350 MPa). In addition to the measure of *F*, the axial reaction at the clamp (equal to the configurational force *M*^2^/2*B* when symmetry applies) was measured with a OC-K5U-C3-R.C. 50N load cell (from Gefran), as well as the midspan deflection *w* (with the displacement transducer fixed at the load frame) and the length Δ*l* (measuring the amount of the rod slipping into the sleeve), with a magnetic non-contact displacement transducer GC-MK5 (from Gemac). Data have been acquired with NI compactRIO system interfaced with Labview 2013 (from National Instruments).

The sleeve in which the rod was free to slide consists of two parts of different length: the lower part (1250 mm) works as a support for the whole polycarbonate strip modelling the elastic rod, whereas the upper part (500 mm), is shorter, so that the magnetic displacement transducer can be accommodated. The lower and the upper surfaces of the sliding sleeve were made using 82 and 32 roller bearings (from Misumi Europe, Press-Fit Straight Type, 20 mm in diameter and 25 mm in length), respectively.

**Figure 7.**
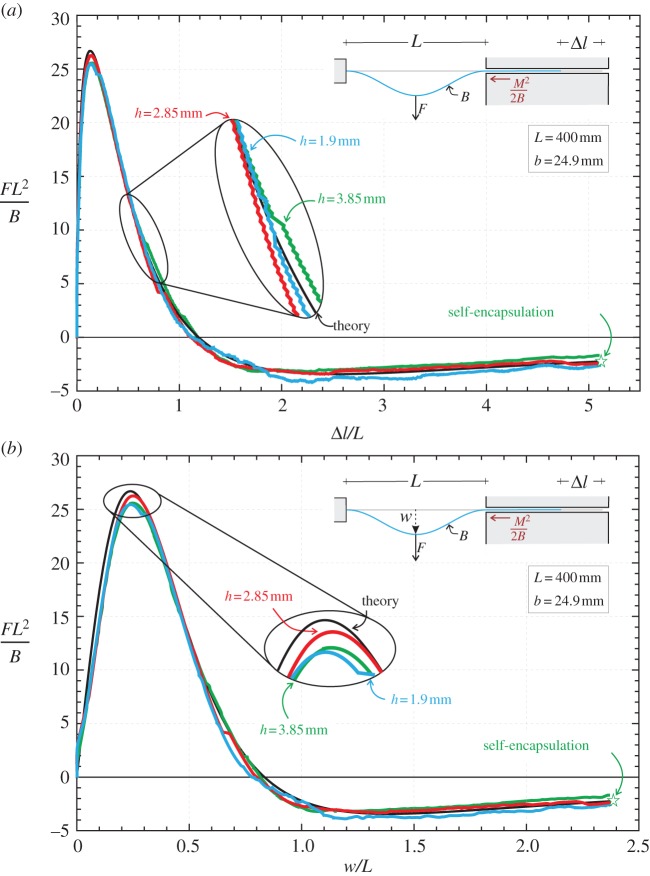
Dimensionless length Δ*l*/*L* measuring the amount of rod slipping into the sliding sleeve (*a*) and dimensionless midspan deflection *w*/*L* (*b*) as functions of the dimensionless transversal load *FL*^2^/*B*: comparison between theoretical (black curve) and experimental results performed on three rods differing only in the thickness *h*, *h*={1.9;2.85;3.85} mm (reported as blue, red and green curves, respectively). The dripping point is marked. Symmetry breaking was observed to occur at the force reversal, but the influence on the measured forces is negligible.

Three photos taken during an experiment, performed on an elastic rod of cross section 24.9×3.85 mm, are reported in figure 6. Experimental results (reported for different thicknesses of the cross section) are presented in figure 7 in terms of dimensionless applied forces versus the amount of rod slipping into the sliding sleeve (figure 7*a*) and the midspan dimensionless deflection (figure 7*b*).

**Figure 8.**
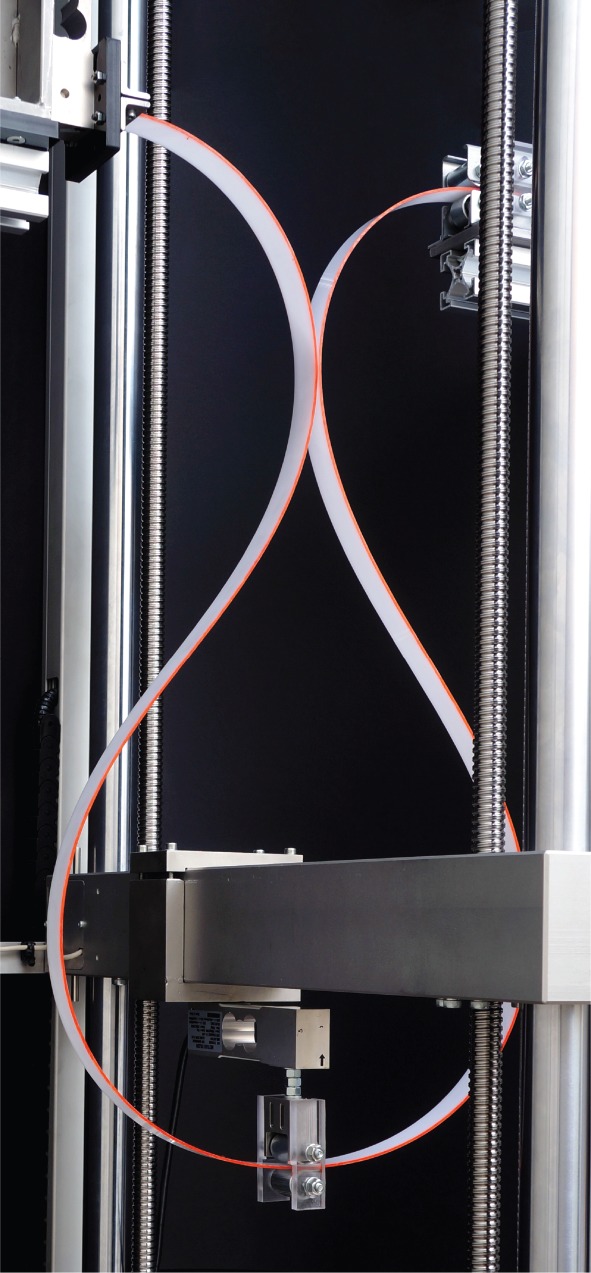
A photo taken at the dripping point during a quasi-static experiment. A symmetry breaking has occurred, although it is not particularly evident.

The experiments were run up to the dripping point and beyond, through the dripping process (where the analytical solution presented in §2 is no longer valid). A photo of an experiment at the dripping point is reported in figure 8. Here a symmetry breaking was encountered at the force inversion point and was found to grow until and after dripping. However, the lack of symmetry was so small that it is hardly visible in the photo.

**Figure 9.**
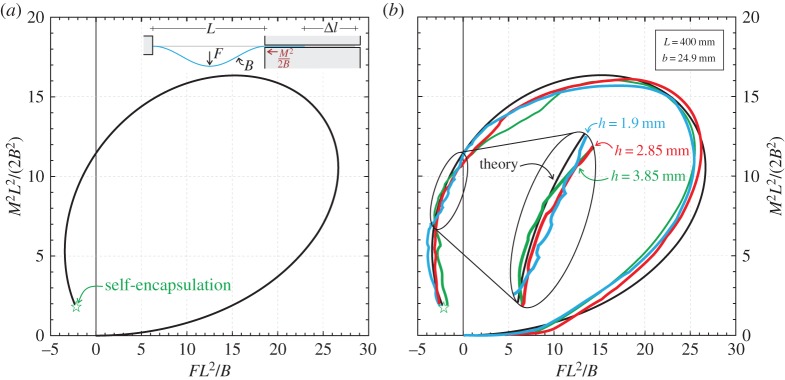
Dimensionless ‘Eshelby-like’ force *M*^2^*L*^2^/(2*B*^2^) versus dimensionless transverse load *FL*^2^/*B*. (*a*) Theoretical solution; (*b*) comparison between theoretical prediction (black curve) and experimental results performed on three rods differing only in the thickness *h*, *h*={1.9;2.85;3.85} mm (reported as blue, red and green curves, respectively). Note the self-encapsulation or dripping point.

Finally, the configurational force *M*^2^/(2*B*) is reported in figure 9 as a function of the transverse force *F* (both forces have been made dimensionless), until the dripping point. The theoretical solution (figure 9*a*) shows that the Eshelby-like force can be much higher than the transverse and dominates the mechanics of the system, as confirmed by the experimental results (figure 9*b*). Moreover, there are regions in the graph that show that the configurational force increases when the applied transverse force decreases.

In conclusion, the comparison between theoretical and experimental results is excellent and shows the following features.

(i) In all experiments a symmetry breaking was observed to occur after the force reversal; however, this lack of symmetry has been found not to preclude self-encapsulation and to be practically negligible on all measured data.
(ii) The shape of the predicted symmetric elastica closely resembles that which is visible in the experiments until symmetry is preserved.
(iii) The experimental load/displacement curve is close to experimental results.
(iv) The predicted load maximum *F*^max^, force reversal at Δ*l*/*L*∼1.19 (or equivalently *w*/*L*∼0.83) and softening are all fully validated by the experiments.

### (b) Dynamic experiments

**Figure 10.**
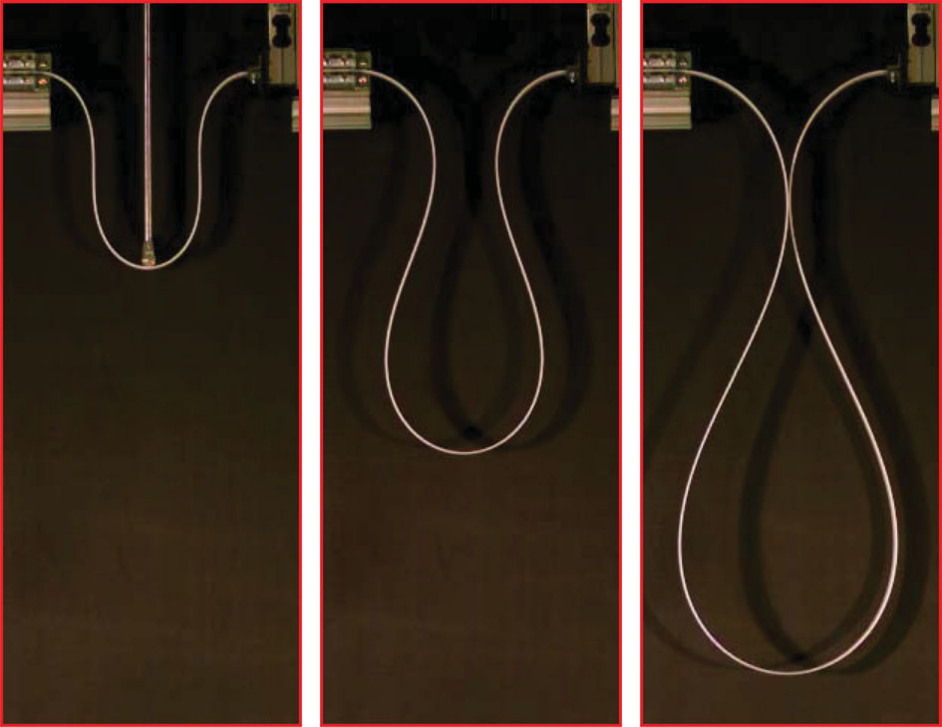
The dripping of an elastic rod during a dynamic experiment. A series of three photos taken with a Photron Fastcam SA5 model 775K-C2 at 500 fps.

Qualitative experiments were performed to definitely substantiate the occurrence of self-encapsulation under dynamic conditions. In these experiments, the rod was brought to the point of load reversal, where an unstable deformed configuration can be maintained at null external load (*F*=0, corresponding to $\hat{\theta }=90^{\circ }$). From here an ambient perturbation is always enough to trigger a dynamics (in which the mass of the rod plays a role) in which the system spontaneously reaches the dripping point and goes beyond this showing a thickening of the drop. The experiments were filmed with a Photron Fastcam SA5 model 775K-C2 at 500 fps and one is presented in the electronic supplementary material. A sequence of photos taken during a dynamic experiment is shown in figure 10 and can be compared with the analogous photos taken (with a Nikon D200 mounting a AF Nikkor 18–35 mm) during a quasi-static experiment, figure 3 (lower part). The comparison between the shapes of the elastic rod during the quasi-static and dynamic experiments shows that dynamics does not have much effect on the results and that symmetry is preserved until the self-encapsulation point is reached.

Finally, although the theoretical results are valid only until self-encapsulation occurs, the quasi-static and dynamic experiments were continued after this instant. Quasi-static and dynamic experiments showed symmetry breaking without sensible deviations from the results obtained under the symmetry assumption. Evidence of multiple self-contacts was neither found, nor expected to occur, while self-intersecting elastica with multiple intersection points can be envisaged, to be checked with a special experimental setting, not considered here.

## 4. Conclusion

The problem of self-encapsulation, or ‘dripping’, of an elastic rod has been posed, solved and validated through quasi-static and dynamic experiments. The results provide a new insight into the possible design of innovative fabrication micro- and nano-technologies based on structural folding.

## Footnotes

1 It is crucial in the statement of the self-encapsulation problem that the constraints are kept at a fixed distance and that the force is transverse, otherwise the problem becomes trivial. In fact, it is well known that in the problem of the buckling of the Euler rod on two movable pins self-intersecting elastica occur, which is also the case of the problem of the ‘injection’ of an elastic rod analysed in [1].

2 Experiments were obtained on structural models designed and realized at the Instability Lab of the University of Trento (http://ssmg.unitn.it).

## Data accessibility

Calculations were performed using Wolfram's MATHEMATICA v. 10. Experimental data are shown in figures 8 and 9 and in the electronic supplementary material.
